# Development and validation of the health-related quality of life instrument for Chinese infertile couples: a mixed-methods study protocol

**DOI:** 10.1186/s12955-022-01957-3

**Published:** 2022-03-28

**Authors:** Zhao Shi, Zhuxin Mao, Hongwei Nie, Ling Geng, Gang Chen, Shunping Li

**Affiliations:** 1 Centre for Health Management and Policy Research, School of Public Health, Cheeloo College of Medicine, Shandong University, Jinan, 250012 China; 2 NHC Key Lab of Health Economics and Policy Research, Shandong University, Jinan, 250012 China; 3 Center for Health Preference Research, Shandong University, Jinan, 250012 China; 4 School of Insurance, Southwestern University of Finance and Economics, Chengdu, 611130 China; 5 Center for Reproductive Medicine, Cheeloo College of Medicine, Shandong University, Jinan, 250001 China; 6 Centre for Health Economics, Monash Business School, Monash University, Melbourne, 3145 Australia

**Keywords:** Infertile couples, Health-related quality of life, Instrument, Validation, Chinese, Protocol

## Abstract

**Background:**

Infertility and its treatment have negative impacts on a couple’s marital relationship, sexual life, psychological state and interpersonal relationships, causing personal distress. Health-related quality of life (HRQoL) has become an important component of health outcomes. HRQoL instruments developed in western culture are not always appropriate for use in China due to cultural differences. Probably due to the unique concept of fertility in China, infertility patients can be looked down upon and the family may  feel shameful. This study aims to develop a HRQoL instrument for infertile couples based on the Chinese social and cultural setting.

**Methods:**

Complementary mixed methods will be used to develop a new HRQoL instrument for Chinese infertile couples. The study consists of four stages: the first stage will involve a systematic review and qualitative interviews to construct draft candidate items. In the second stage, quantitative research [e.g., exploratory factor analysis (EFA), item response theory (IRT)] and cognitive interviews will be used for item selection. The third stage will be instrument validation, in which classical test theory (CTT) and IRT will be applied. In the final stage, the minimal clinically important difference (MCID) will be calculated by using distribution-based methods and anchor-based methods (e.g., logistic regression, receiver operating characteristic curve).

**Discussion:**

The new HRQoL instrument for Chinese infertile couples will be developed, which will provide a standard and effective HRQoL instrument in clinical outcome assessment and health outcome measurement.

## Background

Infertility is defined as the inability to conceive after at least 12 months of unprotected intercourse [1–3]. The infertility rate of couples of childbearing age in China has risen from 2.5 to 3% to around 12 to 15% in 20 years, and the number of patients has exceeded 50 million [4–6]. Infertility and its treatment have negative impacts on a couple’s marital relationship, sexual life, psychological state and interpersonal relationships, causing personal distress [7–9]. With the increasing aging trend in China, infertility has also become an important public health and social problem [4, 5]. In order to raise the fertility level, the three-child policy has been implemented in China [10].

Health-related quality of life (HRQoL) is a multi-dimensional concept that represents the patient’s overall perception of the impact of an illness and its treatment. It includes an individual’s physical, psychological, social aspects of life and has become an important component of health outcomes [11]. Since HRQoL is a culturally relevant concept, people from different cultural backgrounds tend to have different understandings of HRQoL [12, 13]. Western HRQoL instruments are not always appropriate for use in China due to cultural differences between the East and West [13, 14]. It is worth mentioning that there are fundamental differences between Chinese and Western fertility concepts. Infertility patients in China seem to be more influenced by the traditional Chinese saying, “There are three forms of unfilial conduct, of which the worst is to have no descendants” [15], meaning that it is unacceptable for people not to have offspring. Accordingly, infertility patients can be looked down upon and their families may  feel shameful [16, 17]. Women tend to be especially subject to more blame and prejudice than men in the Chinese traditional cultural setting [16].

Disease-specific HRQoL instruments have been developed among infertility patients in previous studies [9, 18, 19]. The fertility quality of life (FertiQoL) scale is currently the most commonly used disease-specific HRQoL scale for infertile couples [18, 20]. However, empirical studies have shown that some items in the FertiQoL are poorly understood in a Chinese cultural setting and irrelevant to the Chinese health service system [21]. Generic HRQoL instruments such as EuroQol-5 dimensions-5 levels (EQ-5D-5L), Medical Outcomes Study 36-Item Health Survey (SF-36), WHO Quality of Life Measure (WHOQOL-BREF) have been frequently used to assess HRQoL among infertility patients [9, 22, 23]. However, the generic measures are not able to include important disease-specific domains, which are useful to detect clinical changes specific to infertility. For example, when EQ-5D-5L was used to measure the HRQoL of infertile patients in China, it was found that the health dimensions of EQ-5D-5L were insufficient, causing a seriously high “ceiling effect” [23].

To the best of our knowledge, there was  only one infertility quality of life instrument developed in China [24]. It focused on those female patients with liver depression and was  based on the theory of traditional Chinese medicine, which limited their application scope. More importantly, the development of the instrument did not include qualitative interviews with infertility patients.

### Objectives

This study aims to develop a de novo HRQoL instrument for infertile couples in China and evaluate its psychometric properties, with the hope of providing a validated HRQoL instrument for the Chinese population.

The specific objectives of the study are :

1. Develop a de novo HRQoL instrument for Chinese infertile couples;
2. Evaluate the reliability, validity, and sensitivity/responsiveness of the instrument;
3. Develop the minimal clinically important difference (MCID) of the instrument and provide thresholds for the interpretation of the results.

## Methods

### Research design

In this study, the new HRQoL instrument development process will refer to the patient-reported outcome (PRO) measures development guidelines recommended by the United States Food and Drug Administration (FDA) [11]. The item identification and domain construction of the instrument will refer to the Wilson-Cleary revised model of HRQoL theory [25, 26], a mixed methods (quantitative and qualitative) design will be used.

**Fig. 1:**
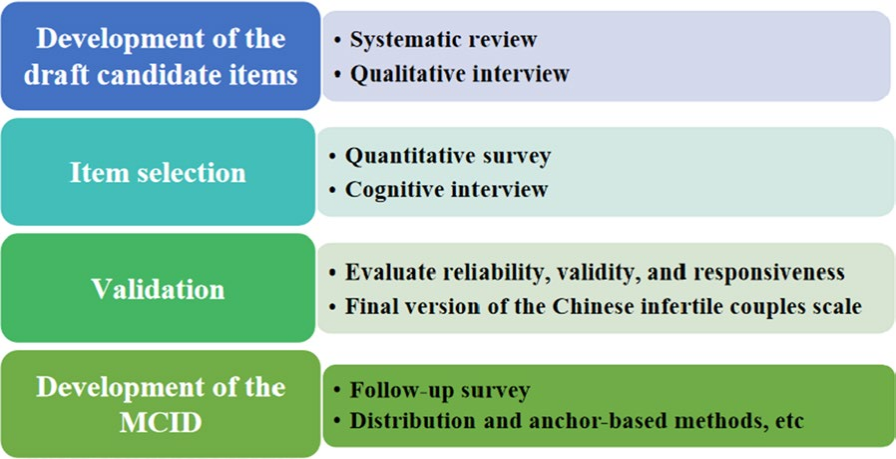
The development process of Chinese infertile couples HRQoL instrument

This study consists of four stages to be implemented sequentially. In the first stage, the item pool will be constructed using a top-down approach based on a systematic review, supplemented with a bottom-up method based on qualitative interviews between clinicians and infertility patients [27]. In the second stage of the item selection, classical test theory (CTT) [e.g., exploratory factor analysis (EFA)] and item response theory (IRT) will be used to select items [28]. Meanwhile, cognitive interviews will be used to test the rationality and comprehensiveness of the items. In the third stage of instrument validation, CTT and IRT will be used to evaluate psychometric properties [28]. In the final stage, the MCID of the HRQoL instrument for infertility patients will be developed in combination with clinical data by using anchor-based methods and distribution-based methods (e.g., logistic regression, receiver operating characteristic (ROC) curve) [28, 29]. The development process of Chinese infertile couples HRQoL instrument is shown in Fig. 1.

### Study samples and recruitment

**Fig. 2:**
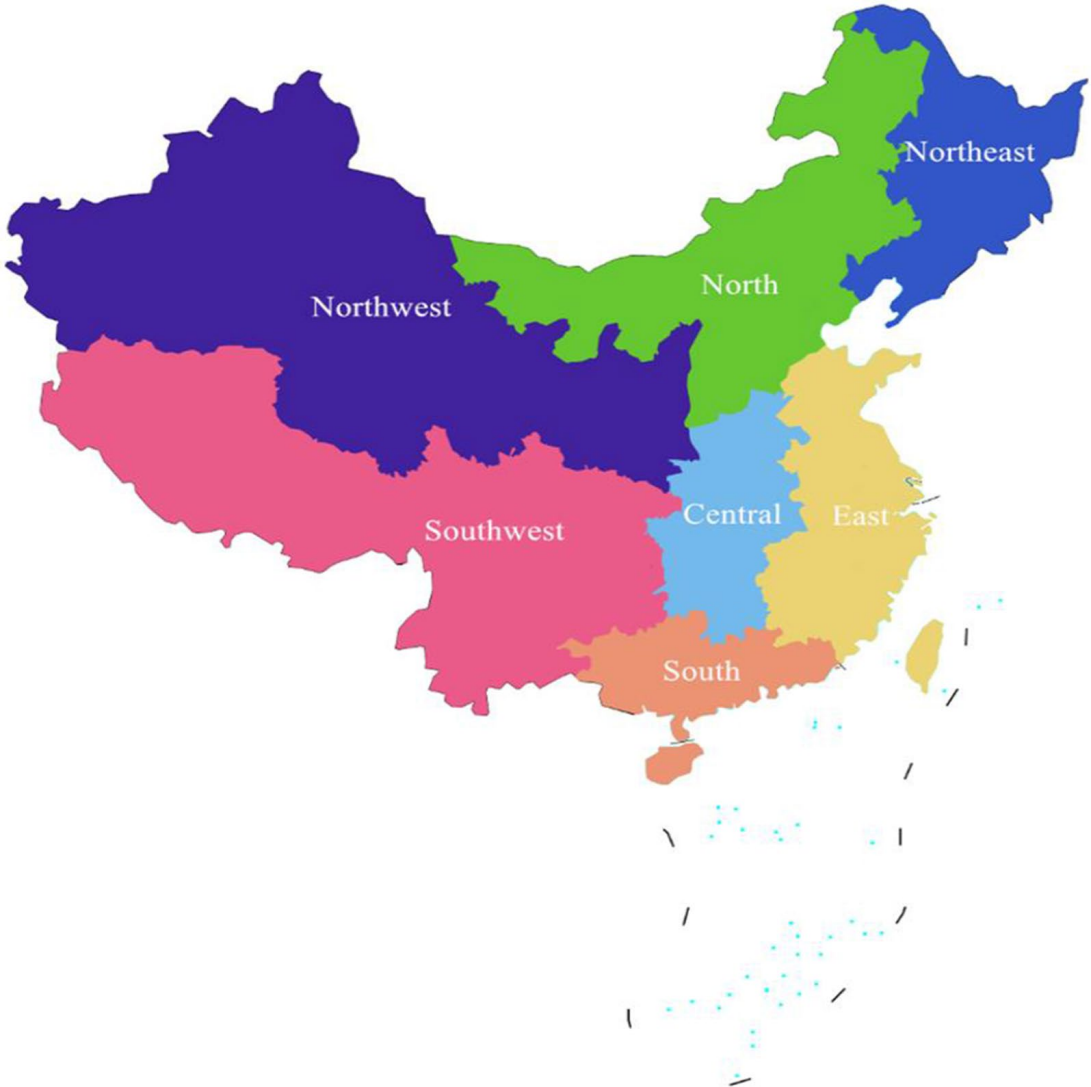
The seven reproductive medicine centers will be selected in China

One reproductive medicine center will be selected in each of the seven regions of the Northeast, Northwest, North, Central, East, Southwest and South China according to geographical location and economic development level in China (Fig. 2). Convenience sampling and purposeful sampling methods will be used to select participants, who receive different treatments, including drugs treatment, surgical treatment, artificial insemination (AI), gamete transplantation, in vitro fertilization-embryo transfer (IVF-ET).

The following inclusion criteria will be applied to determine the eligibility of participating patients: (1) being clinically diagnosed with infertility, that is the inability to conceive after at least 12 months of unprotected intercourse, including primary or secondary infertility patients, male and female infertility patients; (2) giving informed consent; (3) without cognitive impairment.

### Stage 1: Draft candidate items

#### Systematic review

The systematic review will identify a wide scope of studies investigating the either disease-specific or generic quality of life of individuals with fertility problems. To identify such studies, eight databases will be searched, including PubMed, Web of Science, Cochrane Library, Embase, and including the most widely used database for articles written in Chinese, China National Knowledge Infrastructure (CNKI), WanFang Data, China Science and Technology Journal Database (CSTJ), and China Biology Medicine disc (CBMdisc). Key words included in the search strategy will include: infertility (sterility); patient-reported outcomes; quality of life (health related quality of life); health state preference; quality-adjusted life year, etc. Search filters will include the language of English and Chinese.

#### Qualitative interview

##### Design and sample

One-to-one semi-structured in-depth interviews will be held in a private room. In order to diversify the sample, the interviewees will be selected by epidemiological characteristics, such as gender, age group, the determinism of etiology [5, 30], and social demographic characteristics, such as education level [31]. Ten patients will be initially recruited in the Hospital for Reproductive Medicine Affiliated of Shandong University for pilot testing to evaluate whether the patients can understand the key questions of the interview protocol and can respond to the questions. The interview guide will be revised accordingly based on the patients’ feedback. It’s estimated that a total of about 70 infertility patients will be interviewed in seven reproductive medicine centers (Fig. 2). Furthermore, it is estimated that one gynecological clinician, male clinician and psychology/nursing experts will be randomly selected to conduct qualitative interviews at each reproductive medicine center. Three experienced qualitative interviewers will be trained to be competent to undertake semi-structured interviews on clinical reproductive medicine and sensitive topics in seven reproductive medicine centers. An interview guide will be developed to ensure consistency throughout the process.

*Zhihu* is an open access and knowledge-sharing Chinese online community. It contains a large number of electronic diaries of patients’ treatment experiences and psychological processes. Considering that fertility is a relatively private topic, the infertility patient’s electronic diary recorded on *Zhihu* will be used as additional and supplementary material to qualitative interviews [32]. This will provide a more comprehensive view of the problems encountered by infertility patients. Two researchers will use a manual search to sort out and filter the HRQoL information reported by the infertility patient on *Zhihu*, and thematic analysis will be used.

##### Data analysis plan

All interviewers will be transcribed verbatim and subject to thematic analysis based on grounded theory [33–35]. Two independent interviewers will perform the coding analysis, and discrepancies will be  resolved by consensus meetings with the third interviewer to achieve consensus. The coding is mainly divided into three steps [35]. (1) Open coding: two researchers extract information from the transcript, which will be labeled and encoded respectively; (2) Axial coding: classify initial concepts according to relevance and frequency, and aggregate concepts reflecting an aspect together to develop items and domains; (3) Selective coding: the researchers constructed a HRQoL framework for infertility patients by further analyzing the logical relationships among the main items and domains, which will refer to the Wilson-Cleary revised model [26]. Finally, qualitative analysis data will be presented in the word cloud and hierarchy chart. These analyses will be performed in Chinese and then translated into English for reporting. The qualitative analysis will be conducted in MAXQDA 2020 software.

#### Producing draft candidate items

The items identified from the systematic review and the qualitative interview analysis will be used to construct draft candidate items, which will reflect the full range of patients’ experiences and views. The statements (rows) of the interviewees and item concepts (columns) of a systematic review or qualitative interview form a frame matrix by Excel. The patients’ qualitative data will be analysed to produce themes, which will be used to construct draft candidate items. The matrix will help finalise the themes [36]. We will conduct a preliminary test in a small group, with 15–20 patients who do not previously participate, and ask them to comment on the draft candidate items. The draft candidate items will be revised based on their comments to ensure that patients can understand the items and domains.

### Stage 2: Item selection

#### Quantitative surveying

This study adopts methods of convenience sampling and purposeful sampling in quantitative surveying. The questionnaire survey will be implemented among infertile couples from seven reproductive medicine centers, and a face-to-face investigation will be conducted. According to the Graded Response Model (GRM) in IRT, the minimum sample size is calculated to be 500 [37]. We will select 100 infertility patients from each reproductive medical center, and the total number of patients being investigated is expected to be 700. The quantitative survey will consist of socio-demographic information (e.g., age, gender, occupation, annual household income, etc.), clinical characteristics (e.g., infertility duration, type of infertility, treatment history, etc.), the draft candidate items and the FertiQoL instrument.

#### Data analysis plan

Cronbach’s α coefficient, coefficient of variation (CV), Spearman’s rank correlation coefficients, EFA will be used for item selection [28]. The Cronbach’s α coefficient of 0.7 or above will be considered as appropriate [28, 38]. The items of CV greater than 0.25 are considered appropriate. The strength of the correlation (*r*) was interpreted as follows: *r* > 0.7 indicates strong; 0.3 < *r* < 0.7 indicates moderate; *r* < 0.3 indicates weak [39]. EFA will be conducted to select items and examine domains. Factors will be retained if their eigenvalues are greater than 1.0, following the Kaiser (1960) rule [40]. The minimum factor loading of 0.4 will be considered necessary to warrant retaining a statement [41]. In the IRT, Samejima Graded Response Model, which is suitable for multiple response levels [42, 43], will be used to calculate the size of the information function of the item for selection.

#### Cognitive interview

Qualitative feedback regarding the final candidate items will be sought from an expert committee, which is composed of reproductive medicine clinicians, health statisticians, HRQoL researchers and infertility psychological counselling service providers. In addition, cognitive interviews will be conducted among 10–12 patients, who are not previously involved to test the rationality and comprehensiveness of the final candidate items. The patients will be recruited from outpatient clinics and inpatient wards in the Hospital for Reproductive Medicine Affiliated of Shandong University. Furthermore, convenience sampling and purposeful sampling methods will be used to select participants. The previously two experienced qualitative interviewers will conduct the cognitive interview.

### Stage 3: Validation

#### Design and sample

The reliability, validity and sensitivity/responsiveness of the final version of the instrument will be evaluated. We will invite 10–15 experts from reproductive medicine clinical, health statisticians, HRQoL and infertility psychological counseling to evaluate the content validity of the instrument. Convenience sampling and purposeful sampling methods will be used to select participants from outpatient clinics and inpatient wards, who receive different treatments, including drugs treatment, surgical treatment, artificial insemination (AI), gamete transplantation and in vitro fertilization-embryo transfer (IVF-ET). An expected number of 1,400 patients will be investigated in the seven reproductive medicine centers at baseline survey. The electronic or paper questionnaires will be used for face-to-face survey by trained research staff in outpatient clinics and inpatient wards. The questionnaire survey will consist of socio-demographic information (e.g., age, gender, occupation, annual household income), clinical characteristics (e.g., infertility duration, type of infertility, treatment history), the final version of the new instrument and FertiQoL instrument. To evaluate test–retest reliability, a random sample of 200 infertility patients will be selected to complete the instrument again in electronic format online 2 weeks (approximately 10–15 days) after the first test. Most infertility treatment cycles last approximately 2 months through consultation with a reproductive medicine clinician, and HRQoL is expected to change. In order to measure the responsiveness, a follow-up survey will be conducted on all patients who completed the baseline survey to finish the final instrument again after 2 months, and the online surveys will be used.

#### Item and psychometric analysis

##### Reliability

Cronbach’s α coefficient will be applied to estimate the internal consistency reliability, and the intraclass correlation coefficient (ICC) will be used to estimate the test–retest reliability (Table 1). The Cronbach’s α coefficient and ICC of 0.7 or above will be considered appropriate [28, 38].

**Table 1:** Psychometric properties of the HRQoL instrument for Chinese infertile couples

Measurement property	Type	Measure
Reliability	Internal consistency	Cronbach’s α
	Test-rest	Intraclass correlation coefficient (ICC) will be measured at baseline and at 2-week follow-up
	Information function	Information function of each item and domain calculated according to item response theory (IRT)
Validity	Content validity	Item-level content validity index (I-CVI)/Scale-level content validity index (S-CVI)
	Construct validity	Explore factor analysis (EFA); Confirmatory factor analysis (CFA)
	Convergent/discriminant validity	Spearman correlation coefficients
Sensitivity/responsiveness	–	Floor and ceiling effects; effect size (ES); standardized response mean (SRM); paired *t* test

##### Validity

The content validity will be evaluated with item-level content validity index (I-CVI) and scale-level content validity index (S-CVI) based on experts’ ratings of item relevance [44, 45]. EFA and confirmatory factor analysis (CFA) will be used to show the construct validity. EFA will be used to explore the latent factor structure and account for potential correlations among factors. Moreover, CFA will be conducted to test the dimensionality of the de novo instrument and validate the hypothetical structures. FertiQoL is currently the most commonly used disease-specific HRQoL scale for infertile couples [18], which will be used as a reference instrument in construct validity. The association between the Chinese infertile couples HRQoL instrument and the FertiQoL will be studied by using Spearman’s rank correlation coefficients. Convergent validity will be supported if the similar attributes items correlate (*r*) of 0.4 or above, while weak correlations (*r* ≤ 0.3) between dissimilar attributes will support discriminant validity [28].

##### Sensitivity/responsiveness

Sensitivity is the ability to detect differences between groups, responsiveness is the ability of a scale to detect changes [28]. Cohen effect size (ES) and standardised response mean (SRM) will be calculated to evaluate sensitivity and responsiveness, and the paired *t* test will be used to estimate the responsiveness between baseline and 2 months follow up. The same thresholds of 0.2, 0.5 and 0.8 are commonly interpreted for both ES and SRM. An ES or SRM of 0.20 will be considered as small, a value between 0.2 and 0.5 as moderate, and one of 0.8 or greater as large [28]. Floor and ceiling effects can be used as a proxy of sensitivity in relation to how well a measure can detect changes within cross-sectional studies [46]. In this study, floor or ceiling effects will be considered as high if more than 15% of the responders achieve the lowest or highest possible score respectively [47, 48].

### Stage 4: Develop the minimal clinically important difference (MCID)

MCID can be useful to specify a benchmark for interpreting mean differences. It has become a standard approach in the interpretation of clinical relevance of changes in HRQoL [49, 50]. All patients will complete the questionnaires and a 7-point global impression item as an external anchor at the baseline and 2 months follow up. The FertiQoL will be used as an external criterion against which changes in de novo infertility HRQoL instrument will be anchored and calibrated. There are a variety of methods discovering concordance to confirm the choice of an MCID [51]. In this study, we will use distribution-based methods and anchor-based methods (e.g., logistic regression, receiver operating characteristic (ROC) curve) to develop the MCID [28, 29].

## Discussion

Infertility and its treatment have negative impacts on a couple’s marital relationship, sexual life, psychological state and interpersonal relationships, causing personal distress [7–9]. Identifying these factors in infertility patients, creating a good medical environment and offering diverse health education can help improve their health. The new HRQoL instrument for Chinese infertile couples will be developed, which will provide a standard and effective HRQoL instrument in clinical outcome assessment and health outcome measurement.

This study has several advantages. Firstly, the new HRQoL instrument for use among Chinese infertile couples will be developed by using a mixed method. MCID development will provide a benchmark for interpreting mean differences. Secondly, infertility patients and reproduction medicine clinicians will be surveyed from seven reproductive medicine centers to diversify the sample as much as possible, according to the geographical location and economic development level in China. It will provide a comprehensive view of problems encountered by infertility patients. Thirdly, CTT and IRT will be used for item selection and validation, which will fill in the gap that the existing infertility HRQoL instruments development were only based on CTT.

This study also has certain weaknesses. First of all, this study will investigate infertility patients treated in hospitals, and there may be some selection bias in the samples. Future studies may evaluate the psychometric properties in a larger population. In addition, some post information on *Zhihu* may have low reliability.

## Data Availability

Not applicable.

## Abbreviations

HRQoL: Health-related quality of life
FertiQoL: Fertility quality of life
EQ-5D-5L: EuroQol-5 dimensions-5 levels
SF-36: Medical Outcomes Study 36-Item Health Survey
WHOQOL-BREF: WHO Quality of Life Measure
MCID: Minimal clinically important difference
PRO: Patient-reported outcome
FDA: Food and Drug Administration
CTT: Classical test theory
EFA: Explore factor analysis
IRT: Item response theory
ROC: Receiver operating characteristic
AI: Artificial insemination
IVF-ET: In vitro fertilization-embryo transfer
CNKI: China National Knowledge Infrastructure
CSTJ: China Science and Technology Journal Database
CBMdisc: China Biology Medicine disc
CV: Coefficient of variation
GRM: Graded Response Model
ICC: Intraclass correlation coefficient
I-CVI: Item-level content validity index
S-CVI: Scale-level content validity index
CFA: Confirmatory factor analysis
ES: Effect size
SRM: Standardized response mean
